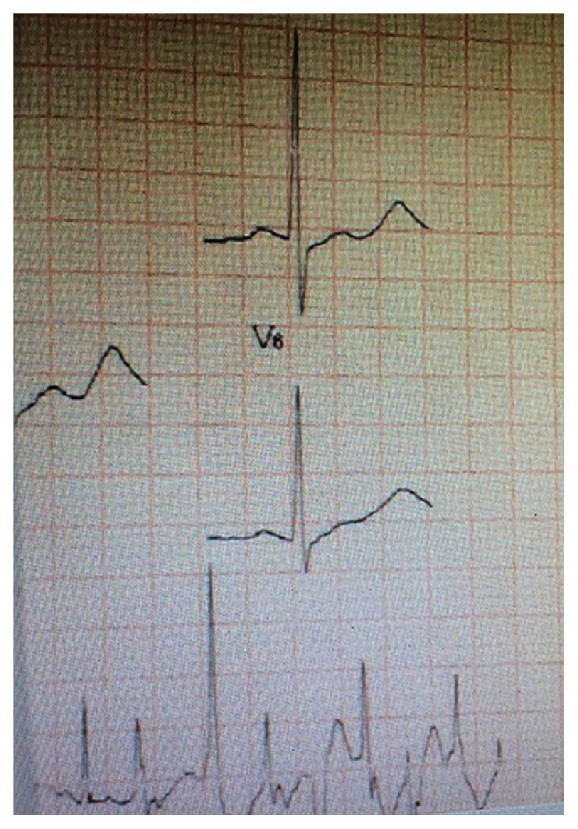# Inconceivable Hypokalemia: A Case Report of Acute Severe Barium Chloride Poisoning

**DOI:** 10.1155/2016/2743134

**Published:** 2016-10-20

**Authors:** Haibo Tao, Yanru Man, Xiaoyuan Shi, Jun Zhu, Hang Pan, Qin Qin, Shanrong Liu

**Affiliations:** Department of Clinical Laboratory, Changhai Hospital, The Second Military Medical University, Shanghai 200433, China

## Abstract

Barium is a heavy divalent alkaline earth metal that has been known as a muscle poison. Barium can cause human toxicity, which may lead to significant hypokalemia and have serious consequences. This paper reports a case of unprecedented barium intoxication in which the patient, who suffered from depression, swallowed at least 3.0 g barium chloride to commit suicide. On admission, the patient presented with nausea, vomiting, stomach burning feeling, dizziness, and weakness. Emergency biochemical testing showed that the patient was suffering from severe hypokalemia (K^+^ 1.7 mmol/L). His electrocardiogram (ECG) prompted atrioventricular blocking, ventricular tachycardia, prolongation of PR interval, ST segment depression with U waves, and T wave inversion. Intravenous potassium supplements were given immediately to correct hypokalemia and regular monitoring of vital signs and fluid balance was arranged. After all-out rescue of our hospital personnel, the condition of the patient is currently stable and he is gradually recovering. This case exemplifies the weaknesses of the management of toxic substances and the lack of mental health education for young people. We hope to get more attention for the supervision of toxic substances and the healthy development of young people.

## 1. Introduction

Barium is found in barite, witherite, and other natural minerals; it has active chemical properties and is easy to be oxidized [1]. Barium and insoluble barium salts, such as barium sulfate, are nontoxic, whereas barium compounds that can dissolve in water or can be diluted in hydrogen acid, including barium chloride, barium carbonate, barium nitrate, barium acetate, and barium sulfate, are toxic. Acute barium poisoning is not commonly seen in clinical practice and this is primarily due to the ingestion of barium salt or food containing barium salt. In addition, soluble barium salt can be absorbed through the skin or inhaled, which makes it difficult to diagnose [2].

It is well known that barium is a muscle poison. It can have an effect on skeletal muscle, smooth muscle, and myocardial excitability and may lead to significant hypokalemia, secondary respiratory paralysis, and malignant arrhythmia, resulting in serious consequences [3]. In general terms of acute barium poisoning, the blood concentration will reach its highest level within 12 h, and symptoms of poisoning will appear within 24 h [4]. Due to the lack of early clinical symptoms, barium poisoning is easy to be misdiagnosed and this has an effect on treatment methodologies. Therefore, timely and accurate diagnosis is crucial for the treatment of acute barium poisoning.

Here we report a case of an unprecedented barium intoxication in which the patient, who suffered from a mild depression, swallowed at least 3.0 g BaCl_2_ that was diluted in 500 mL water to commit suicide. Fortunately, after all-out rescue of our hospital personnel, the patient is currently stable and he is gradually recovering.

## 2. Case Presentation

A 19-year-old male was brought to the emergency department of our hospital with nausea, vomiting, stomach burning feeling, dizziness, weakness, abdominal pain, and diarrhea at 3:00 on April 3rd, 2016. On examination, he was conscious and presented a blood pressure of 120/78 mmHg, a pulse rate of 82 beats/minute, a respiratory rate of 18/minute, and a temperature of 37.2°C. Laboratory testing showed RBC 5.09 × 10^12^/L, WBC 8.10 × 10^9^/L, HGB 152 g/L, PLT 233 × 10^9^/L, K^+^ 2.1 mmol/L, Na^+^ 146 mmol/L, serum glucose (GLU) 5.4 mmol/L, serum creatinine (sCr) 80 *μ*mol/L, ALT 30 U/L, AST 36 U/L, serum amylase (AMY) 97 U/L, total protein (TP) 90 g/L, total calcium (Ca^2+^) 2.75 mmol/L, creatine phosphokinase-MB (CPK-MB) 15 U/L, myoglobin 83.4 ng/mL, and troponin 0.012 ng/mL. All data revealed that the patient presented obvious hypokalemia.

Initially, the physician in charge was unaware that his patient had swallowed barium chloride. After about two hours, the patient showed tongue and facial numbness, tightness in the chest, palpitation, language difficulties, muscle twitches, and unsteady gait. Blood chemistry showed K^+^ 1.7 mmol/L, AMY 128 U/L, total calcium (Ca^2+^) 2.67 mmol/L, CPK-MB 21 U/L, myoglobin 184.2 ng/mL, and troponin 0.021 ng/mL. His ECG showed frequent premature ventricular contractions, atrioventricular blocking, ventricular tachycardia, prolongation of PR interval, ST segment depression with U waves, and T wave inversion (Figure 1). With further deterioration of the patient's condition, he was transferred to the resuscitation room to prepare for the implementation of rescue.

The physicians inadvertently learned that their patient had swallowed abundant barium chloride at about 23:00 on April 2nd, 2016. Immediately, gastric lavage was carried out with 2%~3% sodium sulfate solution and potassium supplementation was intravenously infused at 18.0 mmol/h. In addition, sodium thiosulfate was intravenously administered to reduce the serum concentration of barium ions. During this process, vitamin C, adenosine triphosphate, coenzyme A, glutathione, and liquid were given for treatment and internal environment homeostasis.

After more than two hours of full rescue, the patient's condition was temporarily stable and emergency biochemical testing showed K^+^ 5.8 mmol/L, CPK-MB 24 U/L, myoglobin 115.6 ng/mL, and troponin 0.025 ng/mL. At 17:00, the patient was transferred to an emergency intensive care unit (EICU) for further treatment. In the EICU, potassium correction was continued under strict cardiovascular monitoring and the amount of potassium and speed were continuously adjusted according to urine volume and ECG changes. In the following 24 hours, serum potassium concentration was tested every two hours. Over the next two days, the patient's muscle power improved with rising levels of serum potassium. The patient miraculously made a good recovery without any complications.

## 3. Discussion

Hypokalemia is the most important clinical manifestation of acute barium chloride poisoning. It has been reported that the mechanism of barium ion poisoning resulting in hypokalemia is associated with the ability of the sodium potassium pump (Na^+^-K^+^-ATP enzyme) [5]. The Na^+^-K^+^-ATP enzyme is involved in transmembrane transport of potassium against concentration in intake within the cell. After barium chloride intake, barium ions can increase the activity of Na^+^-K^+^-ATP enzyme and block potassium (K^+^) channels to interfere with passive K^+^ diffusion, leading to the continuous decrease of extracellular potassium [6, 7].

In this particular case, the patient initially did not mention that he had been taking barium chloride, which caused great difficulty in early diagnosis. Because the cause of disease was not clear, the physician was restricted in what he could do. The patient was required to stay in the hospital for further observation. However, his blood potassium level continued to decline, which resulted in severe clinical symptoms. As soon as the physician learned about the barium chloride poisoning, rescue was immediately implemented. Although the time from admission to final diagnosis was approximately ten hours, medical treatment such as gastrolavage, catharsis, and assisted mechanical respiration may improve survival; therefore antidotes, such as soluble sulfate salts that form a precipitate with the barium ions, were administered to gastric lavage or cathartics [8].

Timely, adequate, ultraconventional, multiway to improve the level of serum potassium was the key to success of the rescue. Given that hypokalemia was the primary and lethal pathophysiological factor in barium poisoning that was associated with arrhythmia, respiratory muscle paralysis, and death, intravenous potassium administration was the most effective way to reverse hypokalemia. It is important to ensure that the serum potassium level was increased to 3.0 mmol/L in the initial 3 h and then slow down the speed of potassium supplement [9]. Blood chemistry testing should be performed every two hours to specifically monitor the serum potassium level. In addition, attention should be given to the changes in ECG and urine volume to prevent the high potassium levels.

Due to the lack of specificity in the early clinical symptoms, toxic poisoning as presented in this case is easily misdiagnosed. Combining the large number of barium-related toxicities in the past with clinical manifestations such as muscle paralysis, cardiovascular injury, low potassium levels, and ECG changes, and without symptoms caused by other factors, barium chloride poisoning was finally diagnosed [10]. In this case, we found that the decrease of serum potassium levels increased the levels of myoglobin and troponin, indicating possible myocardial injury. A positive correlation was found between myoglobin, troponin, CPK-MB, and serum potassium levels.

Fatal outcomes due to barium-related poisoning are not uncommon. In the early 1930s, a pattern of illness, termed Pa-Ping, which caused a few deaths in the Sichuan province in China, was observed and investigated. The cause of the disease was due to large-scale food poisoning from the very high proportion of barium chloride in table-salt that was mined in the area [3]. Jiucheng reported that 112 people were poisoned and 5 died after consuming flour containing barium chloride. The blood potassium level of the 5 decedents was less than 2.0 mmol/L and the lower the potassium level, the shorter the incubation period, and the more severe the symptoms [11]. In 1963, two outbreaks of barium poisoning occurred in a large number of people in different villages in Israel [12]. According to the literature, in all cases the patients presented severe and rapid hypokalemia, which was significant and resulted in various clinical effects.

In general, hypokalemia is caused by frequent vomiting, severe diarrhea, too much diuretic, periodic paralysis, and other factors including the loss of potassium, which leads to the ST-T-U changes of ECG and arrhythmia [13]. Low blood potassium levels increase the resting membrane potential up to −90 mv, resulting in the loss of conduction barriers. The characteristic changes in ECG caused by barium poisoning include the activation of Na^+^-K^+^-ATP enzyme and block of K^+^ channels. These changes interfere with passive K^+^ diffusion and may lead to the continuous decrease of extracellular potassium [6]. The ECG revealed frequent premature ventricular contractions, atrioventricular blocking, ventricular tachycardia, prolongation of PR interval, ST segment depression (0.05–0.6 mv) with U waves (up to 1.0 mv), and T wave inversion [4, 14].

Taking into account the clinical features, when a patient shows one of the symptoms shown below, barium poisoning should be considered. It should be noted that those are just initial thoughts: the final diagnosis still needs to be determined:

1. Obvious poisoning symptoms: nausea, vomiting, stomach burning feeling, dizziness, weakness, abdominal pain, and diarrhea
2. Hypokalemia: the serum level of potassium is 1.1–2.8 mmol/L
3. Characteristic changes of ECG: prolongation of PR interval, ST segment depression (0.05–0.6 mv) with U waves (up to 1.0 mv), and T wave inversion
4. Other symptoms: weakness of the limbs, muscle tremor, muscle tension decreased, dyspnea, and arrhythmia among others

In conclusion, this case exemplifies the weaknesses of management of toxic substances and the lack of mental health education for young students. The patient presented in this case is only 19 years old and suffered from a depression, which is not conducive to his healthy development. Therefore, we should strengthen the supervision of toxic substances and enhance safety awareness to avoid the leakage of toxic substances. In addition, we should carry out mental health education on a regular basis to ensure a healthy living environment. Medical professionals should also be aware of the possibility of such accidents and be aware in suspicious cases.

## Figures and Tables

**Figure 1:**